# Sustaining medical research – the role of trust and control

**DOI:** 10.1186/s13561-023-00445-8

**Published:** 2023-05-19

**Authors:** Michael John, Martin Kloyer, Steffen Fleßa

**Affiliations:** 1 Faculty of Law and Economics, University of Greifswald, Friedrich-Loeffler-Str. 70, 17489 Greifswald, Germany; 2 Department of Business Administration, Organization, Human Resources, and Innovation Management, Faculty of Law and Economics, University of Greifswald, Friedrich-Loeffler-Str. 70, 17489 Greifswald, Germany; 3 Department of Business Administration and Health Care Management, Faculty of Law and Economics, University of Greifswald, Friedrich-Loeffler-Str. 70, 17489 Greifswald, Germany

**Keywords:** Control, Expectation of continuity, Interdisciplinarity, Research collaboration, Sustainability, Trust

## Abstract

**Background:**

Medical research is increasingly interdisciplinary. However, not all projects are successful and cooperation is not always sustained beyond the end of funding. This study empirically assesses the effect of control and trust on the sustainability of interdisciplinary medical research in terms of its performance and satisfaction.

**Methods:**

The sample consists of 100 German publicly funded medical research collaborations with scientists from medicine, natural and social sciences (*N* = 364). We develop a system model to analyze the influence of trust and control on performance and satisfaction of the cooperation.

**Findings:**

Both control and trust are important prerequisites for sustainability, control mainly for the performance of the collaboration, and trust primarily for its satisfaction. While the level of interdisciplinarity is a positive moderator for performance, expectation of continuity is a negative intervening variable for the effect of trust and control on satisfaction. Moreover, trust principally adds to the positive impact of control on sustainability.

**Conclusions:**

Interdisciplinary medical research requires a participative but systematic management of the respective consortium.

## Introduction

Medical research is increasingly collaborative work of different disciplines [1]. However, interdisciplinary and collaborative research in medicine as in other subject or organization faces challenges and will not necessarily be more successful than individual research [2]. And in particular, it is not guaranteed that the collaboration will continue beyond the end of the funding period. Instead, many research cooperations will lose momentum even before the project meets its official end as some research partners behave like “predators” [3] with a strong interest in making prey but a low inclination to invest in long-term relations. There is a risk that the cooperation is not performing well, collaboration is not satisfactory and the effort is not sustainable [4].

One reason for low sustainability consists of the different motives of collaborators for joining the consortium. Either, actors cooperate because their own goals correspond to the goals of the cooperation, or they perceive that their goals are more easily achieved by collaborative work [5]. However, it is likely that without credible protective mechanisms, only a collectively suboptimal outcome will result. Nevertheless, these mechanisms of control are difficult to implement in open-ended research processes [6], so that interdisciplinary research also needs to be built on trust [5].

Although there are quite a few studies on interdisciplinarity in research, most of them are not grounded in organizational theory and/or fall short of empirical evidence (e.g. [7–9].), in particular for medical research. We would like to contribute to answering this research question by empirically analyzing consortia in medical research in Germany with the following objectives:

First, we will develop a conceptual model of trust and control in interdisciplinary medical research. Furthermore, we would like to assess empirically the role of these two factors in the sustainability of the medical research consortia. In this context, we define sustainability as the ability of a system to maintain its energy level on a similar scale for a long(er) period [10], i.e., a medical research consortium is sustainable when it continues beyond the end of the specific research agenda or funding.

Second, this research analyses the right mix of trust and control [11]. Although the balance between trust and control seems highly relevant for medical research cooperations, it has to be stated that the relative relevance of trust and control in research cooperations is scarcely studied and controversially discussed in the literature [12]. Only a few authors consider both factors simultaneously, and then mainly as opposites or substitutes (e.g. [13].). However, in line with De Jong and Dirks [14], we assume a supplementary relation of trust and control. We suppose an augmentation effect (booster) of trust that adds to the impact of control on sustainability while the control effect is not replaced but raised by trust.

Third, we will analyze the moderating effect of interdisciplinarity. Interdisciplinarity between different fields of medicine (e.g. surgery, oncology, internal medicine) is a routine, and even research between medical scientists and colleagues from the natural sciences (e.g. microbiology, genetics, etc.) has been strongly developed. However, the collaboration between medical researchers, natural scientists, and counterparts from social sciences (e.g. economists, sociologists, etc.) and humanities (e.g. ethics, linguistics, etc.) is much more difficult and requires more attention. Thus, cooperations can have a different level of interdisciplinarity assuming that the level of interdisciplinarity influences the impact of control and trust. Interdisciplinarity not only helps to deal with complex issues but is also demanding for the actors involved [15].

Finally, this paper will demonstrate the effect of the expectation of continuity of medical research. If a research partner expects that there will be more funding or worthwhile research projects in the future, he/she will likely engage more, i.e., we study the “the performance-enhancing and -diminishing effects” [16] of expectation of continuity as an instrument against opportunism [17].

Our paper is structured as follows. In the next section, we will discuss the methodology of this paper. Afterwards, we will present the findings and discuss their relevance. The paper closes with some recommendations for interdisciplinary medical research.

## Methods

### Conceptional model

**Fig. 1:**
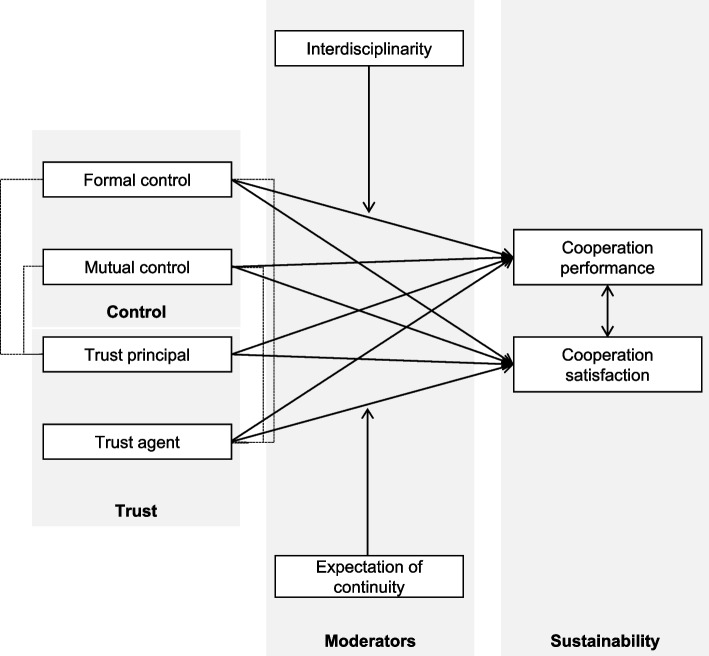
Conceptual framework

Figure 1 gives an overview of our framework explaining sustainability as a result of trust and control. This model is based on the assumption that the balance between trust and control is relevant for all organizations and projects in order to avoid opportunism of individuals or institutions. Also in science, misconduct does exist, for instance in the form of inappropriately assigning authorship credits, plagiarism and using another’s ideas without giving due credit, withholding important details of methodology or results in papers, fraud and falsifying research data and results [18]. Strict control has the potential to reduce opportunism [19], but there is also a risk of hampering innovation, creativity and enthusiasm by a high degree of control. Research is an open-ended and only vaguely structured process, thus formal control is insufficient [6, 20] and would cause high transaction costs [21]. Therefore, trust becomes highly relevant [22] as it reconciles interests, reduces uncertainty, and diminishes transaction costs [20, 23]. Consequently, we have to find the right balance between trust and control in medical interdisciplinary research projects, which is reflected in Fig. 1.

As stated before, a sustainable research project is (here) defined as the continuation of the scientific cooperation beyond the end of the funding period, for instance with second phases, spin-offs, or follow-up projects. The model assumes that research projects can be sustainable if the process of cooperation is satisfactory for the researcher (e.g. good personal relationship, low degree of conflicts, respect, and commitment) and if the performance is high (e.g. publications of high impact factor, achievement of objectives, increased reputation). Consequently, performance and satisfaction are used as proxies for sustainability [24, 25] while there is a positive intercorrelation between the two factors [26, 27].

Control and trust have an impact on performance and satisfaction but have to be analysed in more depth. Firstly, we have to distinguish between formal and mutual control. Formal control describes the incentive of binding regulations and formal agreements, which also facilitate the monitoring of behavior and the measurement of output [11, 12, 28]. Written cooperation agreements are instruments of quality assurance and are often a precondition of public funding. They help to document the obligation for cooperation [5, 9]. Integrated sanctions increase the costs of opportunistic action [29].

Figure 1 assumes the impact of formal control on sustainability. However, non-routine tasks, only vaguely structured processes and open-endedness impede formal control in research [6, 20]. Therefore, in science also mutual control is important [30, 31]. The partners are all peers and continuously evaluate the research process, i.e., they provide the individual members of the consortium with feedback about the performance of the entire cooperation, of certain sub-projects and of individual members of the group [6].

The second component in the balance is trust. Successful collaborative medical research depends on the reliability of one another’s work. Trust helps to coordinate interaction [32, 33] within self-regulating work groups. Because of the embeddedness of actors in collaborative structures, there is no need for relying only on control mechanisms [34]. As risky input [35], trust describes the individual belief not to be treated opportunistically by others [36]. Seeing research cooperations as composed of *n*-principal agent relations [37], it is not only important to get tasks satisfactorily done but also not to be exploited by coworkers. Trustor is not only the principal, trustee not only the agent. To ensure sustainability, it is essential that collaboration partners trust each other both as principal and as agent. Consequently, Fig. 1 distinguishes between the trust of the agent and the trust of the principal which influences the sustainability of the research project.

Furthermore, it is assumed that interdisciplinary and the expectation of continuity have an impact on the strength of the influence of trust and control on sustainability. While the cooperation of researchers from different sciences is essential to deal with complex problems it is also a cultural challenge due to different research traditions, terminology, and values [1, 15]. The more disciplines are involved, the harder the cooperation in research will be, which again lowers performance of and satisfaction with collaboration [5] and, thus, sustainability.

Finally, the model assumes that the anticipation of future cooperation also influences the effectiveness of trust and control. By expanding the former single period model of agency theory by *n* periods actors are embedded in a network of relational contracts [38]. Long lasting relations motivate against opportunism by mutual socioemotional investments [17, 39]. The expectation of continuity is positively related to performance [40] and satisfaction [25].

### Statistical modelling

The conceptual model (see Fig. 1) was formulated as a system equation model and computed with SPSS, version 20. Where available, well-established scales were used. The survey was conducted in German whereas most of the original scales were in English. Consequently, they were translated forward and backward to guarantee content validity. A pretest (*N* = 21) finally checked our measures. Table 1 shows the variables measured.

**Table 1:** Measures

Type of variable	Variable	Sub-variable	Source
Main variable	Sustainability	cooperation performance	[26]
		cooperation satisfaction	[41–43]
	Control	formal control	[44–46]
		mutual control	[47]
	Trust	trust principal	[48]
		trust agent	adopted from [48]
Moderator variables	Interdisciplinarity		own
	expectation of continuity		[49]
Control variables	demographic variables	sex	direct answer
		age	direct answer
		level of education	direct answer
		personal responsibility	direct answer
		experience with interdisciplinarity	direct answer
	network	size of network	direct answer
		proximity of network	direct answer

For both sustainability variables, we calculated 16 regression models (see Table 2). To prevent statistical distortions by using different scales, we z-standardized all measures before calculating. Consequently, our models do not include an intercept. Applying OLS regressions, we also calculated variance inflation factors (VIF). A maximum of 2.50 indicated that multicollinearity is acceptable in our models. Furthermore, we controlled for the common-method bias with Harman’s one-factor test [50] and a non-response bias [51].

**Table 2:** Models

No	Independent variables	interaction	control variables	second sustainability variable	Content
1	-	-	x	-	control model
2	formal control	-	x	-	single effect model with only one antecedent
3	mutual control	-	x	-	single effect model with only one antecedent
4	formal control; mutual control	-	x	-	hierarchical regression model
5	trust principal	-	x	-	single effect model with only one antecedent
6	formal control; trust principal				hierarchical regression model
7	mutual control; trust principal				hierarchical regression model
8	formal control; mutual control; trust principal				hierarchical regression model
9	trust agent	-	x		single effect model with only one antecedent
10	formal control; trust agent				hierarchical regression model
11	mutual control; trust agent				hierarchical regression model
12	formal control mutual control; trust agent				hierarchical regression model
13	all	-	x	-	full model with all predictor variables
14	all	-	x	x	model 13 plus second sustainability variable
15	all	x	x	-	full interaction model with all independent variables and interaction terms
16	all	x	x	x	model 15 plus second sustainability variable

### Data collection and sampling

Our study is initialized by GANI_MED, Greifswald’s approach to individualized medicine, an interdisciplinary research cooperation for establishing efficient personalized medicine, funded by the German Federal Ministry of Education and Research between 2009 and 2014 [52, 53]. In addition to (bio)medical basic research and clinical subjects also actors of economics, philosophy, and theology are involved. To achieve statistical robust results, comparable German medical research cooperations were selected by the funding catalogue of the federal government as the most comprehensive tool to look for German research projects. We did not consider research cooperations for our sample focusing entirely on veterinary medicine, having medicine as a topic without involving medical scientists, being only single and no collaborative project, being mainly privately financed, consisting only of private ventures, not including at least two disciplines, being already institutionalized and/or being located mainly internationally.

## Results

### Sample

The data based offered 305 research consortia, but 67 had to be excluded because they did not fulfill the inclusion criteria (see Sect. 2.3). 138 other consortia did not respond to our mails or did not want to participate. When the coordinator indicated that he/she was willing to contribute to the survey, we asked him/her to provide the link of the survey questionnaire to his/her co-workers. In total, we included 100 research consortia with 2062 researchers. Based on this number, the 364 fully filled questionnaires represent a reply rate of 17.7 20%.

As shown in Table 3, on average 21 researchers of five different disciplines belonged to each consortium, and they worked on average in five different locations. About half of the researchers could be allocated to medical specialties, one third were natural scientists (incl. engineers) and the rest were social scientists. The “average” research group had been cooperating for about four years.

**Table 3:** Sample: basic statistics of consortia (*N* = 100) and Researchers (*N* = 364)

Item	Variable		Median
Consortia	Number of researchers in consortium		20.62
	Number of different disciplines in consortium		4.81
	Interdisciplinarity index (0 = all same discipline)		0.41
	Share of professions [%]	Medicine	42
		Natural Science	32
		Social Science	25
	Number of different locations		4.43
	Current duration of consortium [years]		3.67
	Expected future duration of consortium [years]		1.21
	Total duration of consortium [years]		4.88
Individual Researchers	Age-set of researchers	18–27 years	8.9%
		28–37 years	35.4%
		28–47 years	28.4%
		48–57 years	21.2%
		58–67 years	5.0%
		58–77 years	1.1%
	Highest Education	O-Level	1.4%
		A-Level	2.2%
		Bachelor	3.4%
		Master	37.3%
		PhD	30.3%
		Habilitation/ Junior Professor	5.0%
		Professor	20.4%
	Personnel responsibility		49.2%
	Experience with interdisciplinary collaboration	No experience	33.9%
		1–5 years	30.2%
		6–10 years	19.1%
		11–15 years	9.0%
		16–20 years	5.2%
		> 20 years	2.6%

We asked only research teams which had already been existing and cooperating for some time and which had already started the joint research project. The gender distribution is almost equal and the most frequent age group was between 38 and 47 years with a range of the age-set from 18–27 to 68–77 years. Asked for the educational background, the research groups showed strong differences. While in some groups all researchers were professors (incl. junior professors), some groups had only other academic and non-academic personnel. On average of the entire sample, about one quarter was professor or had the level of habilitation. About half of the respondents was responsible for other members of staff. On average, they had five to six years of experiences with interdisciplinary research consortia.

### Descriptive statistics

Table 1 shows the intercorrelations of our variables together with their means and standard deviations. The statistics indicate that cooperation performance and cooperation satisfaction correlate significantly positive. Highly significant correlations are also found between predictors and criterion variables (and among the control variables themselves). Both control and trust correlate significantly positive with sustainability (Table 4).

**Table 4:** Descriptive statistics (columns 1–7)

	Variables	Mean	S.D	1	2	3	4	5	6	7
1	Cooperation performance	4.23	0.81	1.00						
2	Cooperation satisfaction	4.20	0.94	0.65^***^	1.00					
3	Formal control	3.62	1.04	0.51^***^	0.48^***^	1.00				
4	Mutual control	3.77	0.98	0.44^***^	0.40^***^	0.52^***^	1.00			
5	Trust principal	3.31	1.05	0.29^***^	0.42^***^	0.26^***^	0.21^***^	1.00		
6	Trust agent	4.15	0.82	0.44^***^	0.48^***^	0.30^***^	0.32^***^	0.31^***^	1.00	
7	Interdisciplinarity	3.58	1.04	0.05	0.10^t^	0.09^t^	0.12^*^	0.01	0.02	1.00
8	Expectation continuity	3.45	1.05	0.43^***^	0.57^***^	0.28^***^	0.23^***^	0.24^***^	0.30^***^	0.06
9	Sex	0.50	0.50	-0.09^t^	-0.02	-0.14^**^	-0.13^*^	-0.06	-0.01	0.03
10	Age	2.81	1.09	0.13^*^	0.15^**^	0.05	0.09^t^	-0.06	0.07	0.12^*^
11	Level of education	0.26	0.44	0.16^**^	0.28^***^	-0.01	0.04	0.02	0.08^!^	0.10^!^
12	Personal responsibility	0.49	0.50	0.17^**^	0.17^**^	0.05	0.04	-0.05	0.05	0.05
13	Experience Interdisciplinarity	5.62	6.65	0.12^*^	0.13^*^	0.04	0.08^!^	-0.05	0.12^*^	0.12^*^
14	Size of network	2.90	1.07	-0.13^*^	-0.13^*^	0.05	-0.02	-0.07	-0.22^***^	0.15^**^
15	Proximity of network	3.45	1.20	-0.01	-0.07	0.13^*^	0.06	0.02	-0.02	-0.08

Pearson’s *r*; pairwise deletion; ^!^*p* < 0.20; ^t^*p* < 0.10; ^*^p < 0.05; ^**^p < 0.01; ^***^p < 0.001 (two-tailed)

### Trust and Control

As shown in the full model and in the single effect models (model 13 and models 2 and 3 in Tables 5 and 6) both dimensions of control, formal and mutual control, are positive incentives for the performance of collaboration. The model fit of the full model is significantly improved compared to the control model (model 1). However, sex, age, and personal responsibility are (marginally) significant control variables for the criterion too. Actors, who are female, older, and with personal responsibility judge performance higher. The relevance of both control aspects and all control variables persist when controlling for cooperation satisfaction (model 14).

**Table 5:** Descriptive statistics (columns 8–15)

	Variables	8	9	10	11	12	13	14	15
1	Cooperation performance								
2	Cooperation satisfaction								
3	Formal control								
4	Mutual control								
5	Trust principal								
6	Trust agent								
7	Interdisciplinarity								
8	Expectation continuity	1.00							
9	Sex	0.16^**^	1.00						
10	Age	0.20^***^	0.32^***^	1.00					
11	Level of education	0.35^***^	0.38^***^	0.61^***^	1.00				
12	Personal responsibility	0.30^***^	0.35^***^	0.38^***^	0.54^***^	1.00			
13	Experience Interdisciplinarity	0.26^***^	0.37^***^	0.58^***^	0.63^***^	0.40^***^	1.00		
14	Size of network	-0.16^**^	-0.18^***^	-0.05	-0.05	-0.15^**^	-0.10^t^	1.00	
15	Proximity of network	-0.10^*^	-0.18^**^	-0.05	-0.02	-0.20^***^	-0.13^*^	0.24^***^	1.00

Pearson’s *r*; pairwise deletion; ^!^*p* < 0.20; ^t^*p* < 0.10; ^*^p < 0.05; ^**^p < 0.01; ^***^p < 0.001 (two-tailed)

**Table 6:** Results of regression analyses (standardized regression coefficients ß) with cooperation performance as dependent variable (Model 1–8)

Model	1	2	3	4	5	6	7	8
*Independent variables*								
Formal control		0.50^***^		0.41^***^		0.46^***^		0.38^***^
Mutual control			0.41^***^	0.21^***^			0.36^***^	0.19^***^
Trust principal					0.28^***^	0.16^**^	0.20^***^	0.14^**^
Trust agent								
Interdisciplinarity								
Expectation of continuity								
Cooperation satisfaction								
*Interactions*								
Formal control × Interdisciplinarity								
Mutual control × Interdisciplinarity								
Trust principal × Interdisciplinarity								
Trust agent × Interdisciplinarity								
Formal control × Expectation								
Mutual control × Expectation								
Trust principal × Expectation								
Trust agent × Expectation								
*Control variables*								
Sex	-0.23^***^	-0.15^**^	-0.15^**^	-0.12^*^	-0.23^***^	-0.15^**^	-0.16^**^	-0.13^*^
Age	0.10^!^	0.08^!^	0.06	0.08^!^	0.12^t^	0.09^!^	0.08^!^	0.09^!^
Level of education	0.04	0.07	0.06	0.08^!^	0.03	0.06	0.04	0.07
Personal responsibility	0.14^*^	0.09^!^	0.13^*^	0.10^t^	0.15^*^	0.10^t^	0.14^*^	0.11^*^
Experience with interdisciplinarity	0.07	0.04	0.02	0.02	0.07	0.04	0.03	0.02
Size of network	-0.11^*^	-0.12^*^	-0.09^t^	-0.11^*^	-0.09^t^	-0.11^*^	-0.08^!^	-0.10^*^
Proximity of network	0.02	-0.05	-0.01	-0.04	0.01	-0.05	-0.01	-0.04
*R*^*2*^	0.08	0.32	0.25	0.37	0.16	0.34	0.28	0.38
*R*^*2*^ Change to model 1, 2, 3, 4, 13, resp. 15				0.03^***^		0.02^**^	0.04^***^	0.02^**^
*Adjusted R*^*2*^	0.06	0.31	0.23	0.35	0.14	0.32	0.26	0.36
*N*	333	329	323	320	332	328	322	319

^*!*^*p* < 0.20; ^*t*^*p* < 0.10; ^*^p < 0.05; ^**^p < 0.01; ^***^p < 0.001 (two-tailed)

We assumed that mutual control adds to the effectiveness of formal control. To test this hypothesis we computed a hierarchical regression analysis by introducing formal control firstly into the regression analysis and mutual control secondly. An *F* test was computed to determine any significant improvement of the model fit [54–56]. According to the results of model 4 the augmentation hypothesis is supported.

Tables 7 and 8 show the results of the regression analyses (standardized regression coefficients ß) with cooperation satisfaction as a dependent variable. Based on these statistics we can conclude that both aspects of control are relevant predictors (models 2, 3, 13). The fit of the full model is significantly higher than of the control model (model 1). As a control variable only the level of education is important. As the nonsignificance of the correlations with cooperation satisfaction reveals, the significant effects of sex and proximity of the network are only classical suppressions [57]. In addition, the marginal significance of experience with interdisciplinarity is only a statistical artifact. There is no effect in the control model. The importance of formal control and level of education also exists when controlling for cooperation performance (model 14). Moreover, the augmentation hypothesis can be confirmed. Mutual control positively influences cooperation satisfaction beyond formal control (model 4).

**Table 7:** Results of regression analyses (standardized regression coefficients ß) with cooperation performance as a dependent variable (Model 9–16)

Model	9	10	11	12	13	14	15	16
*Independent variables*								
Formal control		0.42^***^		0.36^***^	0.32^***^	0.22^***^	0.30^***^	0.20^***^
Mutual control			0.30^***^	0.15^**^	0.12^*^	0.08^!^	0.14^*^	0.10^*^
Trust principal					0.06^!^	-0.01	0.06^!^	-0.02
Trust agent	0.43^***^	0.31^***^	0.33^***^	0.27^***^	0.22^***^	0.13^**^	0.22^***^	0.12^*^
Interdisciplinarity					-0.01	-0.02	-0.03	-0.04
Expectation of continuity					0.19^***^	0.04	0.20^***^	0.04
Cooperation satisfaction						0.41^***^		0.45^***^
*Interactions*								
Formal control × Interdisciplinarity							0.09^t^	0.10^*^
Mutual control × Interdisciplinarity							0.05	0.02
Trust principal × Interdisciplinarity							-0.05	-0.09^*^
Trust agent × Interdisciplinarity							0.07^!^	0.08^!^
Formal control × Expectation							-0.07	-0.04
Mutual control × Expectation							-0.03	0.02
Trust principal × Expectation							0.03	0.08^t^
Trust agent × Expectation							0.04	0.06^!^
*Control variables*								
Sex	-0.18^**^	-0.13^**^	-0.14^**^	-0.12^*^	-0.14^**^	-0.11^*^	-0.13^**^	-0.10^*^
Age	0.08^!^	0.08^!^	0.06	0.07^!^	0.09^!^	0.08^!^	0.11^t^	0.08^!^
Level of education	0.02	0.06	0.04	0.07	0.03	-0.03	0.02	-0.05
Personal responsibility	0.16^**^	0.10^t^	0.14^*^	0.11^*^	0.08!	0.08^t^	0.07^!^	0.08^t^
Experience with interdisciplinarity	0.03	0.01	0.00	0.00	-0.01	0.03	-0.02	0.01
Size of network	-0.01	-0.05	-0.02	-0.06	-0.05	-0.05	-0.03	-0.04
Proximity of network	0.02	-0.04	0.00	-0.04	-0.03	0.00	-0.03	-0.01
*R*^*2*^	0.25	0.40	0.33	0.43	0.46	0.52	0.48	0.55
*R*^*2*^ Change to model 1, 2, 3, 4, 13, resp. 15		0.08^***^	0.09^***^	0.06^***^	0.37^***^	0.07^***^		0.07^***^
*Adjusted R*^*2*^	0.23	0.39	0.31	0.41	0.43	0.50	0.45	0.52
*N*	329	325	320	317	314	313	314	313

^*!*^*p* < 0.20; ^*t*^*p* < 0.10; ^*^p < 0.05; ^**^p < 0.01; ^***^p < 0.001 (two-tailed)

**Table 8:** Results of regression analyses (standardized regression coefficients ß) with cooperation satisfaction as a dependent variable (Model 1–8)

Model	1	2	3	4	5	6	7	8
*Independent variables*								
Formal control		0.50^***^		0.40^***^		0.41^***^		0.34^***^
Mutual control			0.40^***^	0.21^***^			0.31^***^	0.17^**^
Trust principal					0.42^***^	0.30^***^	0.35^***^	0.29^***^
Trust agent								
Interdisciplinarity								
Expectation of continuity								
Cooperation performance								
*Interactions*								
Formal control × Interdisciplinarity								
Mutual control × Interdisciplinarity								
Trust principal × Interdisciplinarity								
Trust agent × Interdisciplinarity								
Formal control × Expectation								
Mutual control × Expectation								
Trust principal × Expectation								
Trust agent × Expectation								
*Control variables*								
Sex	-0.16^**^	-0.07^!^	-0.08^!^	-0.04	-0.14^**^	-0.08^!^	-0.08^!^	-0.05
Age	0.04	0.02	0.00	0.01	0.07	0.04	0.03	0.03
Level of education	0.21^**^	0.24^***^	0.23^***^	0.25^***^	0.18^**^	0.21^***^	0.20^**^	0.22^***^
Personal responsibility	0.07	0.03	0.05	0.02	0.10^t^	0.05	0.08^!^	0.05
Experience with interdisciplinarity	0.01	-0.02	-0.03	-0.04	0.02	-0.02	-0.02	-0.03
Size of network	-0.10^t^	-0.10^*^	-0.07^!^	-0.09^t^	-0.07^!^	-0.08^t^	-0.05	-0.07^!^
Proximity of network	-0.05	-0.11^*^	-0.08^!^	-0.11^*^	-0.07	-0.11^*^	-0.08^t^	-0.11^*^
*R*^*2*^	0.09	0.32	0.23	0.36	0.25	0.40	0.35	0.43
*R*^*2*^ Change to model 1, 2, 3, 4, 13, resp. 15				0.03^***^		0.08^***^	0.12^***^	0.08^***^
*Adjusted R*^*2*^	0.07	0.30	0.22	0.34	0.23	0.38	0.33	0.41
*N*	335	331	325	322	333	329	323	320

^*!*^*p* < 0.20; ^*t*^*p* < 0.10; ^*^p < 0.05; ^**^p < 0.01; ^***^p < 0.001 (two-tailed)

As for control, also both trust dimensions are positive antecedents for the performance of collaboration (model 5, 9, 13 in Tables 5 and 6). The importance of trust agent also exists when controlling for cooperation satisfaction (model 14 in Table 6). Trust principal as well as trust agent add significantly to the prediction of the criterion beyond the effect of formal control and/or mutual control. Therefore, augmentation can be confirmed (models 6, 7, 8, 10, 11, 12 in Tables 5 and 6).

For satisfaction with collaboration, trust principal and trust agent are relevant predictors, even when controlling for cooperation performance (models 5, 9, 13, 14 in Tables 7 and 8). Trust principal and trust agent, respectively, significantly improve the model fit of the models involving only formal control and/or mutual control as predictors (models 6, 7, 8, 10, 11, 12 in Tables 7 and 8).

Interdisciplinarity is a relevant moderator of performance. After centering the predictor and intervening variable, the interaction term is generated and all three parameters are regressed on the criterion. Significant interaction terms signal meaningful moderations [58]. The full interaction model (model 15 in Table 6) depicts two (marginally) significant interaction terms, which persist also when controlling for cooperation satisfaction (model 16 in Table 6). Interdisciplinarity affects the impact of formal control and trust agent, respectively, positively. Likewise, the impact of the trust agent on the performance of collaboration is bigger, as the higher interdisciplinarity is individually perceived.

Also for satisfaction interdisciplinarity is a relevant moderator. One significant interaction term is indicated by the full interaction model, which also exists when controlling for cooperation performance (models 15, 16 in Table 8). Interdisciplinarity affects the impact of trust principal positively.

The expectation of continuity is only a relevant moderator for satisfaction with collaboration as criterion. However, expectation of continuity is a highly significant predictor for both sustainability dimensions (model 13 in Tables 6 and 8) and a highly significant correlate of trust and control (Table 3). The full interaction model (model 15 in Table 8) depicts three (marginally) significant interaction terms. The expectation of continuity affects the impact of formal control, mutual control, and trust principal negatively. The moderation with mutual control and trust principal, respectively, persist also when controlling for cooperation performance (model 16 in Tables 8 and 9).

**Table 9:** Results of regression analyses (standardized regression coefficients ß) with cooperation satisfaction as a dependent variable (Model 9–16)

Model	9	10	11	12	13	14	15	16
*Independent variables*								
Formal control		0.39^***^		0.34^***^	0.24^***^	0.13^**^	0.23^***^	0.13^**^
Mutual control			0.28^***^	0.14^**^	0.08^t^	0.05	0.07^!^	0.03
Trust principal					0.19^***^	0.16^***^	0.18^***^	0.16^***^
Trust agent	0.47^***^	0.36^***^	0.38^***^	0.33^***^	0.22^***^	0.15^***^	0.22^***^	0.15^***^
Interdisciplinarity					0.04	0.05^!^	0.04	0.05^!^
Expectation of continuity					0.37^***^	0.31^***^	0.35^***^	0.29^***^
Cooperation performance						0.32^***^		0.32^***^
*Interactions*								
Formal control × Interdisciplinarity							-0.01	-0.04
Mutual control × Interdisciplinarity							0.05	0.03
Trust principal × Interdisciplinarity							0.09^*^	0.11^**^
Trust agent × Interdisciplinarity							-0.01	-0.04
Formal control × Expectation							-0.07^!^	-0.05
Mutual control × Expectation							-0.10^*^	-0.08^t^
Trust principal × Expectation							-0.10^*^	-0.11^**^
Trust agent × Expectation							-0.05	-0.06^!^
*Control variables*								
Sex	-0.10^t^	-0.05	-0.06	-0.04	-0.08^t^	-0.03	-0.07^t^	-0.03
Age	0.02	0.01	-0.01	0.00	0.03	0.00	0.07^!^	0.03
Level of education	0.19^**^	0.23^***^	0.21^***^	0.23^***^	0.15^**^	0.14^**^	0.14^**^	0.13^**^
Personal responsibility	0.10^t^	0.04	0.07^!^	0.04	0.00	-0.03	-0.01	-0.04
Experience with interdisciplinarity	-0.03	-0.05	-0.05	-0.06	-0.07^!^	-0.07^!^	-0.06	-0.06
Size of network	0.01	-0.02	0.01	-0.02	-0.01	0.01	0.02	0.03
Proximity of network	-0.06	-0.11^*^	-0.08^!^	-0.11^*^	-0.07^t^	-0.06^!^	-0.07^t^	-0.06^!^
*R*^*2*^	0.29	0.43	0.35	0.44	0.57	0.63	0.63	0.68
*R*^*2*^ Change to model 1, 2, 3, 4, 13, resp. 15		0.11^***^	0.12^***^	0.09^***^	0.49^***^	0.05^***^		0.05^***^
*Adjusted R*^*2*^	0.27	0.41	0.33	0.42	0.56	0.61	0.60	0.66
*N*	331	327	322	319	315	313	315	313

^*!*^*p* < 0.20; ^*t*^*p* < 0.10; ^*^p < 0.05; ^**^p < 0.01; ^***^p < 0.001 (two-tailed)

## Discussion

### Trust and control in balance

The main goal of our study was the empirically based extension of present research on the efficient ratio between control and trust and its meaning for sustainability of interdisciplinary medical research. Sustainability was therefore differentiated in performance of and satisfaction with collaboration. The intercorrelation of both dimensions is significantly positive. For performance mainly control is important. On the contrary, trust is of prime relevance for satisfaction. Together with only a correlation of *r* < 0.70, the different antecedent focus supports our concept of the bi-dimensionality of sustainability. Moreover, trust adds to sustainability beyond control.

Both results, the bi-dimensionality of sustainability and the augmentation effect, are important new insights into interdisciplinary medical research and therefore help to build a systematic theory of interdisciplinarity in medical research. The positive effects of trust and control are no trivial findings, as their impact on performance and satisfaction is far from consistent. Positive, negative, and even no significant effects are documented in the literature [14, 59].

Furthermore, the sample might have an impact on trust and control as gender, age and professional status are important determinants of cooperation success. In our analysis we realized that researchers who are female, older and with personal responsibility have a tendency to assess the effectiveness of the research consortium more positively. Furthermore, the educational status of a researcher has an impact on his satisfaction with the cooperation. Professors showed to be more satisfied with the interdisciplinary research consortium and its performance than researchers of a lower status. Whether the sample was representative for all interdisciplinary research groups in Germany and internationally could not be analysed, i.e., the sample might lead to a bias.

### Moderation by interdisciplinarity and expectation of continuity

While interdisciplinarity is a positive moderator of the effect of trust and control on performance, expectation of continuity is a negative moderator with satisfaction as criterion variable.

The level of interdisciplinarity is strictly positive for sustainability. Distinct thematic research profiles with not (too) incompatible working routines may be beneficial for medical research groups. A major rationale for research cooperations is the expansion of research capacity and the need for complementary expertise [60]. While authors like Dewulf, François [15] see cooperation in research as more difficult the more diverse the disciplines involved are in terms of cognition, methodology, and structure, Pelz [61] already explained half a century ago that scientists benefit most when interacting with others from dissimilar backgrounds while at the same time exchange ideas with at least one important colleague with similar professional values. Both, purely disciplinary organized reward systems and predominantly disciplinary constituted structures rather minimize than encourage competition within interdisciplinary settings. Scientific careers are made inside disciplines, not across them [1]. Moreover, complementary research topics prevent situations of direct competition in interdisciplinarity. That again facilitates cooperation [5].

The impact of trust and control on satisfaction with collaboration is always negatively moderated by expectations of continuity. This result is quite surprising for trust, as it seems to contradict the well-established concept of the “shadow of the future” ([17], p. 124) and its meaning for building trust and cooperation. As Deeds and Hill [62] already pointed out, relational contracting needs not inevitably protect against opportunism, since in “an extended series of exchange interactions” there is also “the chance for miscommunication or misinterpretation of each other’s action or motives” ([63], p. 148). Initial commitment may give way to distrust. Additionally, the extension of time horizon may be accompanied by an increasing irrelevance of trust to ensure sustainability. The perceived security that there will be collaboration in the future too (e.g., by establishing new research institutes) means that one’s own qualifications are negligible. External structures and constraints relieve actors from trusting others to work efficiently with them.

### Managerial implications

Besides only ad hoc generated guidelines, theoretically founded and empirically validated concepts are important in order to ensure sustainable collaboration in interdisciplinary research in academia. In contrast to overly optimistic contributions that generally expect “a climate of trust “ in research ([5], p. 233), our results prove the importance of both, control and trust, to accomplish sustainability.

Control is particularly relevant for performance of collaboration. In contrast to the risk of control for crowding out motivation, we found no such effect. However, it is important to differentiate, because trust is more important than control for satisfaction with collaboration. Moreover, trust adds to the positive impact of control on sustainability (augmentation). Trust may increase voluntary compliance, commitment to collective goals, and willingness to exhibit extra-role behavior [11, 59]. In that respect, monitoring and control are less essential [63]. Therefore, both mechanisms must be balanced. Neither to insist only on control, nor solely on trust is quite efficient [64].

Differently to our conceptual model, the expectation of continuity is consistently a negative moderator variable. The impact of both, trust and control, on satisfaction with collaboration is the lesser the more one does expect the research cooperation to continue. Equally surprising, interdisciplinarity always has a positive effect on sustainability. Rather than being only challenging interdisciplinarity is foremost beneficial. Trust and control are most efficient when research cooperations are built from many different scientific disciplines. Confronted with other perspectives one may overcome one’s own scientific limits.

### Limitations 

Our study faces a number of limitations. Firstly, our sample was based on the funding catalogue of the federal government of Germany. Consequently, all research groups were from Germany with a certain institutional set-up and a German research tradition that might not be representative for other countries. Research groups where the majority of researchers could not speak German, were excluded from the analysis. Furthermore, there might have been some non-German speaking respondents within this group, but this was not reflected in our questionnaire although trust and control are cultural values which differ a lot between nationalities [65].

Secondly, our study is based on cross-sectional data. Because results in academic research might need often ten years or more to be presented [66], longitudinal data would be more feasible to assess performance and satisfaction. However, panel mortality necessitated a methodological compromise. No longer being at the beginning all considered research cooperations were already long established. Consequently, causality can only be theoretically checked for plausibility, but not empirically verified. A replication as a longitudinal study would ensure the chronological asymmetry of cause and effect.

Thirdly, due to the complexity of our conceptual model, follow-up surveys with large data sets (*N* > 500) must also be conducted. In particular, the effect of negative moderation by expectation of continuity, which seems to contradict the positive meaning of lasting relationships for cooperation and trust, needs further studies.

Finally, it would be interesting to check if our results apply to all actors involved in medical research cooperations. According to previous research, there are some indications of different types of actors in interdisciplinary research [67, 68], but more research is needed to understand the balance of trust and control in collaborative medical research.

## Conclusion

Medical research is increasingly interdisciplinary extending far beyond the traditional boundaries of medicine, i.e., medical research consortia include natural scientists (e.g. micro biologists, geneticists, physicists, chemists), but more and more also colleagues from social sciences (e.g. sociologists, economists) and the humanities (e.g. philosophers, linguists). This interdisciplinarity is enriching, opens the door to new and fruitful research and is often the only solution to handle complex and comprehensive research topics of relevance for the society. Growing research cooperations between medical specialists and other subjects also requires a common understanding of values, a similar language of communication (which is frequently not too easy between different scientists, even when they speak the same mother tongue) and a code-of-conduct. In other words, we need some form of management of the medical research consortium.

However, this management cannot be based on strict control, since there is no single person or group of people who has the right to give instructions to others. Instead, research calls for an innovative, open and respectful mode of collaboration based on trust. However, trust alone might not be sufficient, so medical research cooperations require the right balance of trust and control.

We have shown that trust and control are equally important to sustain research cooperations. However, our paper also demonstrates that it is necessary to differentiate between two facets of sustainability. Control is essential for the performance of collaboration, while trust is most crucial for the satisfaction of collaboration. Trust also adds to the positive effect of formal and/or mutual control (augmentation effect). Moreover, interdisciplinarity and expectation of continuity are relevant moderators. The impact of trust and control on sustainability is bigger when interdisciplinarity is high, while expectation of continuity is low.

Consequently, interdisciplinary medical research benefits from a balance of trust and control. It all starts with trust in the colleagues and their integrity. This is the door-opener for a joint project and innovative thinking. The better we know each other and the more time and effort we invest in getting to know each other, the better it is for the sustainability of the project. However, trust must not be blind. As soon as a person in a research consortium behaves opportunistically, control measures must be in place to identify the misconduct at an early stage and to initiate countermeasures [69]. A good starting point is when principal investigators and project leaders see themselves not only as scientists alone, but also as managers of the consortium with the obligation to improve the cohesion of the consortium, balance trust and control, and together developing the vision for beneficial interdisciplinary research.

## Data Availability

All data generated or analyzed during this study can be obtained from the authors.
